# Absorbable polymeric surgical clips for appendicular stump closure: A randomized control trial of laparoscopic appendectomy with lapro-clips

**DOI:** 10.18632/oncotarget.9283

**Published:** 2016-05-11

**Authors:** Bo Lv, Xin Zhang, Jun Li, Shusheng Leng, Shuqiang Li, Yunlong Zeng, Bing Wang, Jiatian Yuan, Jun Fan, Shasha Xing, Ci Li

**Affiliations:** ^1^ General Surgery Department, Affiliated Hospital/Clinical Medical College of Chengdu University, Chengdu, 610081, P.R. China; ^2^ Central Laboratory, Affiliated Hospital/Clinical Medical College of Chengdu University, Chengdu, 610081, P.R. China; ^3^ Pathology Department, Affiliated Hospital/Clinical Medical College of Chengdu University, Chengdu, 610081, P.R. China

**Keywords:** absorbable clip, appendicitis, appendix stump closure, laparoscopic appendectomy

## Abstract

A randomized control trial was performed to evaluate the effectiveness and safety of absorbable polymeric clips for appendicular stump closure in laparoscopic appendectomy (LA). Patients were randomly enrolled into an experimental group (ligation of the appendicular base with Lapro-Clips, L-C group) or control group (ligation of the appendicular base with Hem-o-lok Clips, H-C group). We identified 1,100 patients who underwent LA between April 1, 2012 and February 3, 2015. Overall, 99 patients (9.0%, 99/1,100) developed a complication following LA (47 [8.5%] in the L-C group and 52 [9.5%] in the H-C group (*P* = 0.598). No statistically significant differences were observed in intra-abdominal abscesses, stump leakage, superficial wound infections, post-operative abdominal pain, overall adverse events, or the duration of the operations and hospital stays between the groups (all *p* > 0.05). Adverse risk factors associated with the use of absorbable clips in LA included body mass index ≥ 27.5 kg/m^2^, diabetes, American Society of Anesthesiologists degree ≥ III, gangrenous appendicitis, severe inflammation of the appendix base, appendix perforation, and the absence of peritoneal drainage. The results indicate that the Lapro-Clip is a safe and effective device for closing the appendicular stump in LA in select patients with appendicitis.

## INTRODUCTION

Laparoscopic appendectomy (LA) is frequently performed to treat appendicitis [1]. It is a well-established surgical technique that has several advantages compared to open appendectomy (OA) such as a faster recovery time and reduced rate of infection [2, 3, 4]. LA is currently the standard treatment for appendectomy at many institutions. In fact, many studies have demonstrated that LA reduces surgical time, results in shorter hospital stays, and lowers the rate of complications compared to OA [2–7]. A variety of techniques have been used to ligate the appendicular stump, which is a critical step in LA to prevent infection including endostaples, endoloops, and non-absorbable metal or polymeric ligation clips [8–11]. In our study, the use of absorbable polymeric clips (Lapro-Clips), which are widely used in laparoscopic cholecystectomy, was evaluated. We assessed the feasibility and safety of Lapro-Clips compared to non-absorbable polymeric clips (Hem-o-lok Clips) in a large number of patients who underwent LA.

## RESULTS

A total of 1,100 patients were recruited to our study between April 2012 and February 2015. Of these patients, 550 received Lapro-Clips to ligate the appendicular base (L-C group), while the other 550 patients received Hem-o-lok Clips to ligate the stump (H-C group). There were no significant differences in patient age, gender, body mass index (BMI), or American Society of Anesthesiologists (ASA) degree between the two groups (Table 1). Nine surgeons participated in the study.

**Table 1:** Clinical characteristics of patients in the L-C and H-C groups

Variable	L-C Group (*n* = 550)		H-C Group (*n* = 550)		X^2^	*P*
	NO.	%	NO.	%		
Mean age ± SD, years	37.4 ± 15.3		36.8 ± 16.1		1.313^a^	0.239
Gender						
Female	294	53.5	301	54.7	0.179	0.672
Male	256	46.5	249	45.3		
BMI, kg/m^2^						
< 23.0	260	47.3	280	50.9	1.475	0.478
23.0–27.5	182	33.1	171	31.1		
≥ 27.5	108	19.6	99	18		
Diabetes						
No	480	87.3	493	89.6	1.504	0.220
Yes	70	12.7	57	10.4		
ASA degree						
I	271	49.3	266	48.4	0.119	0.730
II	230	41.8	240	43.6		
III	47	8.5	43	7.8		
IV	2	0.4	1	0.2		

Abbreviations: SD, standard deviation; BMI, body mass index; ASA, American Society of Anesthesiologists;

a *T*-test result.

### Overall adverse events

There were 122 adverse events observed in the L-C group and 139 in the H-C group (*p* = 0.228). Overall adverse events included all complications, re-interventions, and other adverse events (e.g., enteritis, persistent elevated inflammatory parameters, sepsis with methicillin-resistant staphylococcus aureus, persistent diarrhea, intra-abdominal fluid collection, and constipation). No significant differences in other adverse events were identified between the L-C and H-C groups (4.2% vs. 4.9%, *P* = 0.563). Finally, no patients died as a consequence of either surgical approach.

### Intra-operative and histological findings

In the L-C group, the average diameter of the appendix base was 8.52 ± 2.03 mm (range 3.5–11.0). In contrast, the average diameter in the H-C group was 8.27 ± 2.13 mm (range 3–10) (*P* = 0.326). There were no significant differences in the category of appendicitis, degree of inflammation of the base of the appendix, perforation (not in the base), peritoneal drainage, or histology (*P* > 0.05) between the two groups. All of these data are presented in Table 2.

**Table 2:** Patient intra-operative and histological findings in the L-C and H-C groups

Variable	L-C Group (*n* = 550)		H-C Group (*n* = 550)		X^2^	*P*
	NO.	%	NO.	%		
Size of appendix base (mean ± SD, range, mm)	8.52 ± 2.03 (3.5–11.0)		8.27 ± 2.13 (3–11.0)		1.136^a^	0.326
Appendicitis						
Pure	311	56.5	298	54.2	0.902	0.637
Purulent	184	33.5	199	36.2		
Gangrenous	55	10.0	53	9.6		
Inflammation of appendix base						
0 (none)	97	17.6	105	19.1	0.106	0.744
1 (slight)	264	48.0	257	46.7		
2 (moderate)	159	28.9	153	27.8		
3 (severe)	30	5.5	38	6.9		
Perforation (not in the base)						
No	415	75.5	398	72.4	1.362	0.243
Yes	135	24.5	152	27.6		
Peritoneal drainage						
No	354	64.4	370	67.3	1.034	0.309
Yes	196	35.6	180	32.7		

Abbreviation:

a *T*-test result.

### Complications

A total of 99 patients (9.0%, 99/1,100) developed a complication after LA (47 [8.5%] in the L-C group and 52 [9.5%] in the H-C group (*P* = 0.598). An intra-abdominal abscess (IAA) developed in 29 patients (5.3%) in the L-C group and 33 patients (6.0%) in the H-C group (*P* = 0.179). No statistically significant differences were observed in stump leakage, superficial wound infection, or post-operative abdominal pain between the two groups (all *P* > 0.05) (Table 3). No patients experienced bleeding after surgery.

**Table 3:** Outcomes of patients in the L-C and H-C groups

Variable	L-C Group (*n* = 550)		H-C Group (*n* = 550)		X^2^	*P*
	NO.	%	NO.	%		
No. of clip						
1	470	85.5	453	82.4	1.946	0.163
2	80	14.5	97	17.6		
Overall complications	47	8.5	52	9.5	0.278	0.598
IAA	29	5.3	33	6.0	1.807	0.179
Stump leak	1	0.2	2	0.4	0.334	0.563
Superficial wound infection	14	2.5	12	2.2	0.158	0.691
Post-operative abdominal pain	3	0.5	5	0.9	0.504	0.478
Overall re-intervention	52	9.5	60	10.9	0.636	0.425
Percutaneous and/or transrectal drainage	3	0.5	2	0.4	0.201	0.654
Re-operation	11	2.0	13	2.4	0.17	0.680
Prolonged intravenous antibiotics	33	6.0	41	7.5	0.927	0.336
Readmissions	5	0.9	4	0.7	0.112	0.738
Others^a^	23	4.2	27	4.9	0.335	0.563
Overall adverse events	122	22.2	139	25.3	1.452	0.228
Duration of operation ± SD, min	41.4 ± 19.8		40.6 ± 20.3		1.012^b^	0.679
Duration of hospital stay ± SD, days	3.56 ± 2.11		3.49 ± 2.33		1.135^b^	0.531

Abbreviations:

a other adverse events including enteritis, persistent elevated inflammatory parameters, sepsis with MRSA, persistent diarrhea, intra-abdominal fluid collection, and constipation;

b *T*-test result.

### Re-interventions

Re-interventions were required in 52 patients (9.5%) in L-C group compared to 60 patients (10.9%) in H-C group (*P* = 0.425). A total of 11 patients (2.0%) in the L-C group underwent additional surgery because of IAA or stump leakage, while 13 patients (2.4%) underwent additional surgery in the H-C group (*P* = 0.680). No statistically significant differences were observed in percutaneous and/or transrectal drainage, prolonged administration of intravenous antibiotics, or readmission between the two groups (*P* > 0.05).

### Duration of surgery and length of hospital stay

The average duration of surgery (from the time the incision was made to the time of skin closure) was 41.4 ± 19.8 min in the L-C group and 40.6 ± 20.3 min in the H-C group (*P* = 0.679). The mean length of hospital stay was 3.56 ± 2.11 days in the L-C group and 3.49 ± 2.33 days in the H-C group (*P* = 0.531). There were no significant differences in the duration of surgery or the length of hospital stay between the two groups.

### Risk factors for adverse events

The frequency of all adverse events among all patients was 261. In order to evaluate risk factors for adverse events, we compiled the data for all 1,100 patients. Using Chi-squared tests, we determined that BMI, diabetes, the category of appendicitis, inflammation of the appendix base, ASA degree, perforation of the appendix (not in the base), and peritoneal drainage impacted the incidence of adverse events (*P* < 0.05). Gender did not influence the prognosis of patients with appendicitis following surgery (*P* = 0.980). Peritoneal drainage could effectively reduce the risk of adverse events. According to the statistical analyses, one clip was safe for appendicular stump closure (rate of adverse events: 23.1% in the L-C group vs. 27.1% in the H-C group, *P* = 0.247) (Table 4).

**Table 4:** Analysis of risk factors for LA-associated adverse events for all patients

Variable	All Patients (*n* = 1100)		All Adverse Events (*n* = 261)		X^2^	*p*
	No.	%	Frequency	%		
Gender						
Female	595	54.1	141	23.7	0.001	0.98
Male	505	45.9	120	23.8		
BMI, kg/m^2^						
< 27.5 (normal and overweight)	893	81.2	199	22.3	5.459	0.019
≥ 27.5 (obese)	207	18.8	62	30.0		
Diabetes						
No	973	88.5	180	18.5	116.372	< 0.001
Yes	127	11.5	81	63.8		
ASA degree						
I, II	1007	91.5	196	19.5	119.635	< 0.001
III, IV	93	8.5	65	69.9		
Appendicitis						
Pure	609	55.4	115	18.9	52.043	< 0.001
Purulent	383	34.8	91	23.8		
Gangrene	108	9.8	55	50.9		
Inflammation of the appendix base						
0–1 (none, slight)	723	65.7	142	19.6	28.137	< 0.001
2 (moderate)	312	28.4	88	28.2		
3 (severe)	68	6.2	31	45.6		
Perforation (not in the base)						
No	813	73.9	163	20.0	23.293	< 0.001
Yes	287	26.1	98	34.1		
No. of clip						
1	923	83.9	213	23.1	1.341	0.247
2	177	16.1	48	27.1		
Peritoneal drainage						
No	724	65.8	200	27.6	17.774	< 0.001
Yes	376	34.2	61	16.2		

## DISCUSSION

LA is currently the preferred technique for the treatment of acute appendicitis. Recent studies have shown that > 50% of appendectomies were performed by laparoscopy [12]. LA has several advantages including a shorter surgery time, shorter hospital stay, faster recovery, and a lower rate of superficial wound infection [2, 3, 4]. Appendicular stump closure is critical in order to avoid infections and complications such as stump leakage and IAA. Endostaplers, endoloops, and endoclips have been used for stump closure in LA. All can be used for adequate stump closure, and each has advantages and disadvantages. There are no general recommendations in the relevant literature at present.

Endostaplers and endoloops have been compared in several studies. For example, Aajid et al. [13] compiled the data from five randomized controlled trials with 622 patients and concluded that endoloops were superior to endostaplers. This was because stump closure with endoloops had a similar complication rate compared to that of endostaplers but had a much lower cost. In contrast, one review indicated that the routine use of endostaplers is preferable, especially in patients with an inflamed appendix base, due to being associated with a lower complication rate compared to endoloops [14]. Another disadvantage of endoloops is that there is a requirement for a sufficient amount of expertise in placing and tightening the loops around the appendix base [15]. Endoclips including non-absorbable polymeric and metal clips are less frequently used in LA, although clips have been used for appendicular stump closure for more than 20 years [16]. The use of endoclips is technically limited because the appendix is inflamed and has a large base. However, several studies have determined that endoclips are feasible and safe to use metal endoclips or non-absorbable polymeric clips to close the appendicular stump in select patients [17, 18, 19].

In past 2 decades, absorbable clips are frequently used in laparoscopic cholecystectomy, and the feasibility and safety of these clips are widely accepted [20]. There are similar standards for using Hem-o-lok Clips and Lapro-Clips including the size and degree of inflammation of the appendix base. Several have demonstrated the feasibility and safety of Hem-o-lok Clips for appendicular stump closure [21, 22]. Hence, we evaluated the effectiveness and safety of Lapro-Clips for appendicular stump closure in LA compared to Hem-o-lok Clips.

Lapro-Clips are advantageous in that they are made of absorbable polymeric materials. The clips will be absorbed by the body within 90 (internal portion)–180 days (external portion). This means there will be no foreign body remaining in the enterocoelia following LA. Metal clips, non-absorbable polymeric clips, and endostaplers may interfere with imaging methods such as X-ray, computed tomography, and magnetic resonance imaging. The absorbable clip potentially provides long-term benefits to patients following surgery because it avoids the impact of foreign bodies. The Lapro-Clip could not be used in patients with a large appendix base (> 11 mm) or severe inflammation in appendicular base. However, in the present study, it can be used in most patient populations. The considerations related to Lapro-Clips use are also applicable to other devices such as endoloops and metal or non-absorbable polymeric clips. However, we found that the Lapro-Clip could also be safely applied in some patients with an inflamed appendix base if the appendix base was only slightly or moderately inflamed. Recent studies have indicated that endoloops can be used in patients with an appendix base ≤ 15 mm, titanium clips in patients with a base of ≤ 20 mm, and Hem-o-lok Clips in patients with a base of ≤ 10 mm [23, 18, 24].

In 14.5% of patients, two clips were used at the appendix stump while in 85.5% of patients, surgeons used only one clip. It was advisable to transfer the appendix immediately into a retrieval bag in order to avoid contamination. We did not find significant differences in adverse events between the single-clip and double-clip groups, which indicated that the use of one clip was sufficiently safe in LA.

Typical complications associated with appendicitis include IAA and stump leakage. Peritoneal drainage reduces the rate of IAA significantly [25, 26, 27]. In this study, the rates of IAA and stump leakage were 5.3% and 0.2% in the L-C group, respectively. These complication rates are comparable to recent studies [28, 29]. The durations of surgery and hospital stays vary significantly depending on the country in which the study was performed. Recent studies have reported shorter operating times (46.3–64.9 min) [9, 23, 30, 31]. In our study, the operation time in our study was 41.4 ± 19.8 min in the L-C group and 40.6 ± 20.3 min in the H-C group. These times were comparable to those of other studies. Several studies have reported a median hospital stay of 2–5.9 days, with no significant differences between the different methods for stump closure [2, 18, 32].

The use of clips instead of staplers has resulted in reduced costs. In our study, the price of one absorbable clip was approximately 274Y ($43.50) and the price of one Hem-o-lok clip was 148.5Y ($23.60). The mean cost of LA was 9362Y ($1,486.00) in the L-C group and 9297Y ($1,475.70) in the H-C group, respectively. The costs of LA in both groups (including the readmission fees) were lower than those reported by other studies [18, 31].

In this study, we evaluated risk factors for both absorbable and non-absorbable polymeric clips in stump closure in LA. Based on the data, we conclude that the risk factors included BMI ≥ 27.5 kg/m^2^, diabetes status, ASA degree ≥ III, gangrenous appendicitis, severe inflammation of the base of the appendix, perforation of the appendix, and the absence of peritoneal drainage, which indicates surgeons should use endostaplers instead of clips in patients with these risk factors to reduce the rate of complications and adverse events.

The limitation of this study was that patients with different category of appendicitis, especially those with IAA and/or gangrene in appendix were enrolled, which may result in higher adverse events and potential bias. The way to make the conclusion more reliable may enroll patients with purulent appendicitis but without gangrenous appendicitis. Besides, although the result indicated that the outcomes are similar in 1 and 2 absorbable clips groups, it may conduct potential bias. Thus, more strict study design is necessary.

## CONCLUSIONS

The Lapro-Clip is a safe and effective device for closing the appendicular base in LA. The absorbable clip shows a comparable complication rate and adverse event rate to the Hem-o-lok Clip. Thus, the Lapro-Clip is a suitable tool for LA that does not result in residual foreign bodies and does not increase the economic burden of patients. There are some limitations of the Lapro-Clip in patients with a severely inflamed appendix and/or a large base. Surgeons should evaluate the safety of using the Lapro-Clip according to the risk factor model.

## MATERIALS AND METHODS

### Study design

**Figure 1:**
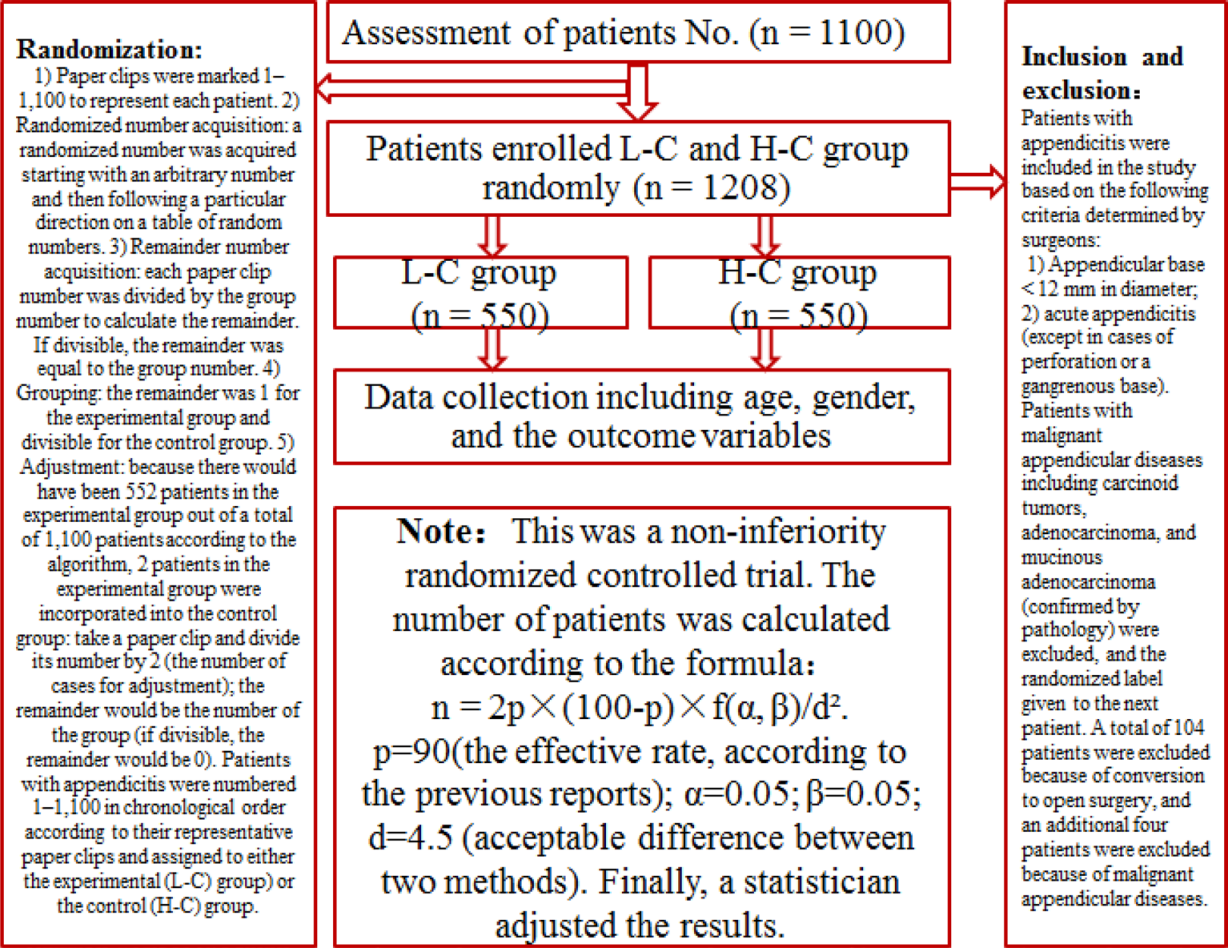
Study design

The study was a randomized control trial. Prior to beginning the study, we planned to randomly recruit 1,100 patients with appendicitis to the patient cohort based on statistical considerations (Figure 1).

### Patients

Patients with appendicitis (pure, purulent or gangrenous) who could meet the inclusion criteria were informed of the approach the surgeons would use to close the appendicular stump and were also told that their data (including pre-operative demographic, intra-operative, and outcome) would be documented in the study. The inclusion and exclusion criteria are shown in Figure 1. If the patients met the inclusion criteria, the surgeon ligated the appendicular stump with either Lapro-Clips or Hem-o-lok Clips depending on the patient group. Additionally, patients with malignant appendicular diseases (including carcinoid tumor, adenocarcinoma, and mucinous adenocarcinoma) confirmed by pathology were excluded, and the randomized label was given to the subsequent patient.

### Surgeons

All surgeons participating in this study could perform appendicular closure with Lapro-Clips or Hem-o-lok Clips proficiently. Participating physicians were instructed to close the appendicular stump with one or two clips based on their own clinical judgment and to submit information related to the parameters and outcomes of the treatment to the database.

### Device descriptions

**Figure 2:**
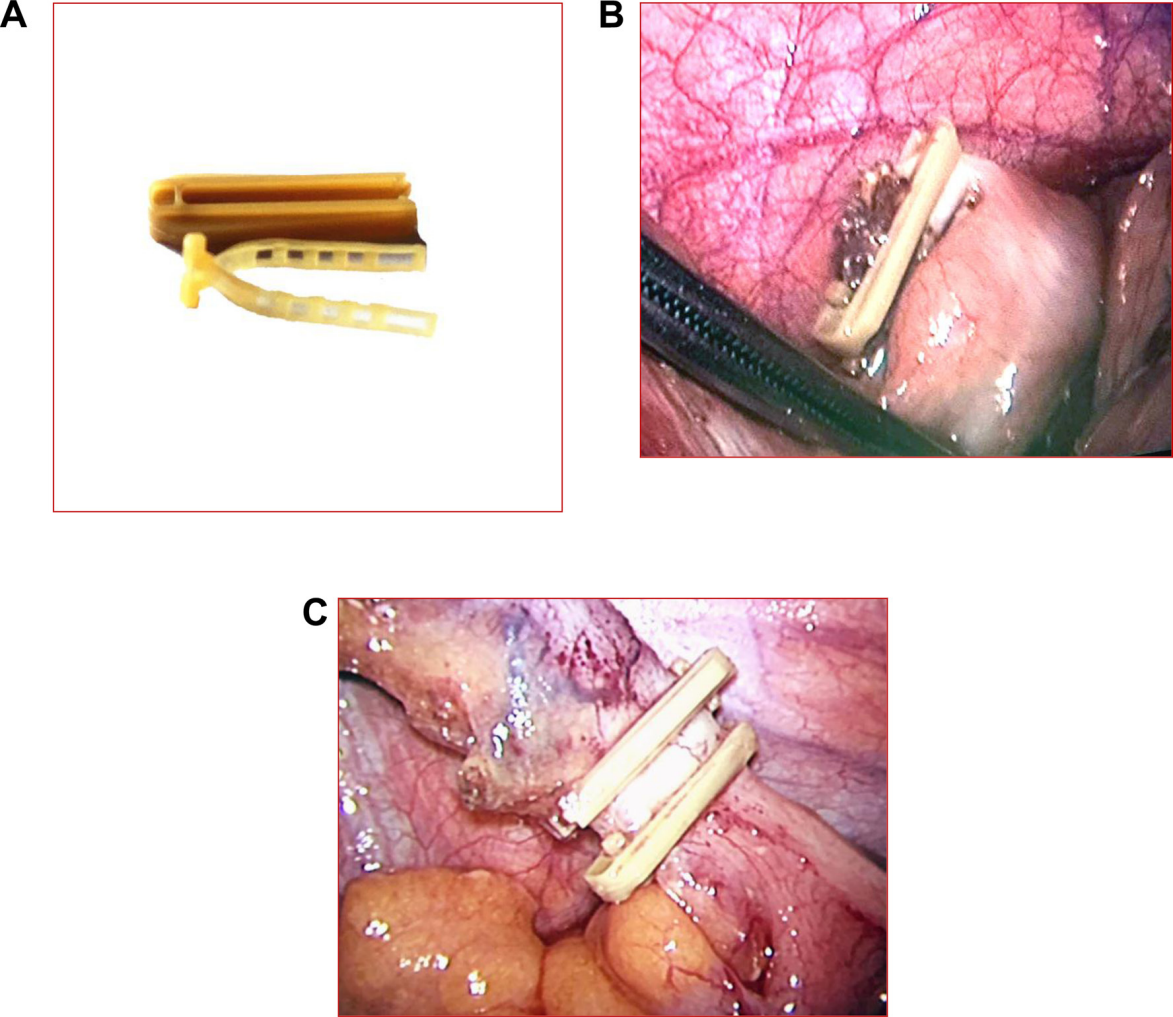
(A) The internal and external portions of the Lapro-Clip The clip can close tissue with a diameter ≤ 12 mm (**B**) One clip for stump closure. (**C**) Two clips for stump closure (all reserved).

The absorbable clip is made of absorbable polymeric materials including poly hydroxy acid and poly glucose ester (the Lapro-Clips, Convidien, NC, USA, 20143465649). The clips are absorbed by the body within 90 days (internal portion) and 180 days (external portion). Lapro-Clip devices are intended for use in laparoscopic cholecystectomy and other procedures in which clips are indicated (Figure 2A).

### Ethical considerations

In this study, the product was used within the indication. Furthermore, no additional examinations or interventions were performed on the patients. Therefore, this study was not considered to constitute an additional risk for enrolled patients. Ethical approval was obtained from the appropriate ethics committees before patients were enrolled in the study. Written informed consent was obtained by the investigator at each center from all patients prior to enrollment.

### Treatments

LA and pre-operative or concomitant treatments were performed according to the standards. LA was performed using the three-port approach. The Lapro-Clip was used according to the manufacturer's instructions. Before the study began, the hospital was informed about the study procedures. Surgeons resected the mesoappendix and the artery of the appendix using an ultrasonic knife without any other management. This knife was also used to remove the appendix. The application of one clip to the stump was recommended and estimated to be sufficient for closure. The surgeon decided whether or not to use an additional clip (Figure 2B, 2C). The surgeons were asked to estimate the degree of inflammation of the appendix base using a scale from 0 (none) to 3 (severe inflammation). Peritoneal drainage was performed if the surgeons estimated the degree of inflammation to be ≥ 3. If conversion to open surgery occurred during an operation, the surgeons closed the stump using an absorbable line instead of two types of clips.

### Outcome variables

The primary outcome variables were the following: 1) Post-operative complications including IAA, superficial wound infection, appendicular stump leakage, and post-operative abdominal pain, which was defined as abdominal complaints after surgery requiring prolonged clinical observation or additional biochemical or radiological tests; 2) Re-interventions including percutaneous and/or transrectal drainage, re-operation (laparoscopy/laparotomy), and prolonged use of intravenous antibiotics (> 3–5 days); 3) Duration of the operation (the time from skin incision to skin closure), duration of hospital stay, and readmission (the duration of a readmission was included in the hospital stay calculation).

### Follow-up

As follow-up, a telephone interview was conducted 30 days after surgery. Adverse events, re-admissions to the hospital, and medical treatments related to the operation that were required were recorded.

### Statistical analysis

All the data obtained during this study were tabulated and subjected to analysis using the standard methods of statistical analysis. Statistical analyses were performed using SPSS, version 19.0. Chi-squared tests were performed to evaluate proportional differences between the two groups. *T*-tests were performed for continuous data. We considered *p* values < 0.05 to be statistically significant.
